# Silencing Heat Shock Protein 47 (HSP47) in Fibrogenic Precision-Cut Lung Slices: A Surprising Lack of Effects on Fibrogenesis?

**DOI:** 10.3389/fmed.2021.607962

**Published:** 2021-02-15

**Authors:** Mitchel J. R. Ruigrok, Khaled E. M. El Amasi, Diana J. Leeming, Jannie M. B. Sand, Henderik W. Frijlink, Wouter L. J. Hinrichs, Peter Olinga

**Affiliations:** ^1^Department of Pharmaceutical Technology and Biopharmacy, Groningen Research Institute of Pharmacy, University of Groningen, Groningen, Netherlands; ^2^Nordic Bioscience, Herlev, Denmark

**Keywords:** collagen chaperone, Gp46, gene silencing, HSP-47, lung explant culture, lung fibrosis, RNA interference

## Abstract

Idiopathic pulmonary fibrosis (IPF) is a chronic disease that is characterized by the excessive deposition of scar tissue in the lungs. As currently available treatments are unable to restore lung function in patients, there is an urgent medical need for more effective drugs. Developing such drugs, however, is challenging because IPF has a complex pathogenesis. Emerging evidence indicates that heat shock protein 47 (HSP47), which is encoded by the gene *Serpinh1*, may be a suitable therapeutic target as it is required for collagen synthesis. Pharmacological inhibition or knockdown of HSP47 could therefore be a promising approach to treat fibrosis. The objective of this study was to assess the therapeutic potential of *Serpinh1*-targeting small interfering RNA (siRNA) in fibrogenic precision-cut lung slices prepared from murine tissue. To enhance fibrogenesis, slices were cultured for up to 144 h with transforming growth factor β1. Self-deliverable siRNA was used to knockdown mRNA and protein expression, without affecting the viability and morphology of slices. After silencing HSP47, only the secretion of fibronectin was reduced while other aspects of fibrogenesis remained unaffected (e.g., myofibroblast differentiation as well as collagen secretion and deposition). These observations are surprising as others have shown that *Serpinh1*-targeting siRNA suppressed collagen deposition in animals. Further studies are therefore warranted to elucidate downstream effects on fibrosis upon silencing HSP47.

## Introduction

Idiopathic pulmonary fibrosis (IPF) is a chronic and progressive respiratory disease characterized by the pathological deposition of extracellular matrix (ECM) in the lungs (1). As IPF progresses, the lungs gradually lose their ability to facilitate gas exchange, leading to breathlessness and, ultimately, death. Epidemiological studies point toward an incidence of 2-30 cases per 100,000 person years and a prevalence of 10–60 cases per 100,000 people (2). In addition, patients have a poor prognosis because the median survival time after diagnosis has been estimated to be 3 to 5 years. To date, only two drugs (pirfenidone and nintedanib) have been approved for the treatment of IPF. Although pirfenidone and nintedanib do not actually cure IPF, they are often prescribed to slow its progression. These drugs, however, have also been shown to cause serious side effects, such as gastro-intestinal bleeding, diarrhea, and liver toxicity (1). Therefore, there remains an unmet medical need for more effective and safer drugs to treat IPF.

Developing such drugs is challenging because IPF has a complex pathogenesis (3, 4). To minimize toxic effects and to maximize therapeutic effects, drug targets should remain largely confined to diseased tissue and contribute sufficiently to the pathogenesis (5). Myofibroblasts, for example, play a key role in the pathogenesis of IPF and are attractive cells to target as they produce ECM proteins (e.g., collagens and fibronectins) (6). Out of all potential drug targets in myofibroblasts, heat shock protein 47 (HSP47) is particularly interesting because it is essential for the biosynthesis of collagens, such as collagen type 1 (COL1) which is overexpressed in fibrosis (7). In the endoplasmic reticulum (ER), HSP47 facilitates the folding of procollagens into trimeric structures (triple-helices). Subsequent trafficking of these trimeric collagens from the ER to the Golgi apparatus is also mediated by HSP47. Inhibition or knockdown of HSP47 could therefore be a promising strategy to attenuate fibrosis in patients.

So far, research focused on knockdown of HSP47 rather than its pharmacological inhibition (8, 9). One of the major difficulties of developing inhibitors is that compound screening procedures are time-consuming and laborious as active human HSP47 is unstable (8, 10). As an alternative, small interfering RNA (siRNA) can be used to transiently knockdown HSP47 via RNA interference (11). Until now, several animal studies revealed that knockdown of HSP47 ameliorated fibrosis in various models (e.g., renal, peritoneal, pulmonary, hepatic, vocal fold, and skin fibrosis) (12–17). Bleomycin-induced pulmonary fibrosis, for example, was suppressed in rats after intravenous administration of vitamin A coated liposomes containing *Serpinh1*-targeting siRNA (14). Results from this study also suggest that therapeutic effects were attributable not only to reduced collagen deposition but also to apoptosis of myofibroblasts. These findings are encouraging and should be validated in other experimental models and species.

Traditional *in vitro* models (i.e., cell cultures), however, are not suitable for obtaining insights into affected molecular pathways because they lack a relevant biological context and, accordingly, offer limited insights into tissue-wide effects of siRNA-mediated HSP47 knockdown. To that end, precision-cut lung slices are interesting as they are viable explants, with a well-defined thickness and diameter, that can be cultured *ex vivo* for up to a few days (18). A key advantage of this model includes its ability to recapitulate functional and structural features of the lungs, such as the presence of different cell types and the maintenance of cell-cell and cell-matrix interactions. As a result, lung slices can be used to study airway physiology, fibrogenesis, and biotransformation (18). Furthermore, we previously demonstrated that lung slices can be successfully transfected with self-deliverable (Accell) siRNA to trigger specific mRNA and protein knockdown (19, 20). This model can therefore be used to characterize the effects of siRNA-mediated HSP47 knockdown in a biologically relevant environment.

Motivated by the need for more effective and safer drugs to treat IPF, we aimed to investigate the therapeutic potential of *Serpinh1*-targeting siRNA in lung slices prepared from murine tissue. We first confirmed whether fibrogenesis could be augmented in slices by using transforming growth factor β1 (TGFβ1)—a potent pleiotropic cytokine that plays a key role in the development of IPF (4). Various aspects of fibrogenesis were assessed, such as mRNA expression of fibrogenesis-related genes, secretion of fibronectin into culture medium, and expression of alpha smooth muscle actin (α-SMA). Furthermore, because HSP47 is involved in the biosynthesis of collagens, we monitored the secretion of COL1 and its incorporation into the ECM as well as the formation of fibrillar COL1 and collagen type 3 (COL3) networks. After characterizing the effects of TGFβ1 on fibrogenesis, we examined whether *Serpinh1*-targeting Accell siRNA triggered knockdown of *Serpinh1* mRNA and its respective protein HSP47. Finally, we set out to explore whether knockdown of HSP47 affected the development of fibrogenesis as well as the secretion and deposition of collagen.

## Materials and Methods

### Animals

Lungs were collected from male C57BL/6J mice (10–14 weeks old; 24–30 gram), which were maintained under 12 h light/dark cycles, with free access to water and food (Central Animal Facility, University Medical Center Groningen, Groningen, The Netherlands). Mice were first anesthetized with 5% isoflurane/O_2_ gas (Nicolas Piramal, London, UK). Once rendered unconscious, mice were euthanized by exsanguination via the inferior vena cava followed by perforation of the diaphragm. Directly afterwards, the lungs were inflated *in situ* with 1 mL of liquefied and pre-warmed (37°C) support medium consisting of 1.5% low-gelling-temperature agarose (Sigma-Aldrich, Zwijndrecht, The Netherlands) and 0.9% NaCl (Merck, Darmstadt, Germany). After exposing the thoracic cavity, the lungs were excised and immediately placed in ice-cold University of Wisconsin (UW) preservation solution (Dupont Critical Care, Waukegab, USA). The animal experiments were approved by the Central Authority for Scientific Procedures on Animals (permit number: 20171290) and were conducted conform national and international legislation.

### Lung Slice Preparation

After excision of the lungs, lobes were separated from each other and cylindrical tissue cores were prepared with a biopsy puncher. To preserve the viability, tissue cores were immediately transferred to ice-cold UW preservation solution. Slices with a wet weight of 4-5 mg, thickness of 250–350 μm, and diameter of 5 mm were prepared using a Krumdieck tissue slicer (Alabama Research and Development, Munford, USA), which was filled with ice-cold Krebs-Henseleit buffer supplemented with 25 mM D-glucose (Merck), 25 mM NaHCO_3_ (Merck), and 10 mM HEPES (MP Biomedicals, Aurora, USA); saturated with carbogen gas (95% O_2_ and 5% CO_2_); and adjusted to a pH of 7.4 (21). Directly afterwards, slices were transferred to ice-cold UW preservation solution.

### Culturing Lung Slices

After their preparation, slices were either sampled (0 h) or pre-incubated individually in 12-well plates, containing pre-warmed (37°C) culture medium (1 mL/well), at 5% CO_2_ and 20% O_2_ while being horizontally shaken (90 cycles/min). Culture medium was composed of DMEM/F-12 + GlutaMAX™ (Fisher Scientific, Landsmeer, The Netherlands), 100 U/mL penicillin-streptomycin (Life Technologies, Bleiswijk, The Netherlands), and 50 μg/mL gentamicin (Life Technologies). After a pre-incubation of 2 h, slices were transferred to culture plates with fresh and pre-warmed culture medium and they were incubated for either 48, 96, or 144 h. Culture medium was refreshed every 48 h. To augment fibrogenesis, slices were cultured in medium containing 5 ng/mL TGFβ1 (Roche, Basel, Switzerland). In knockdown experiments, slices were incubated without Accell siRNA (untransfected) or in medium with either 0.5 μM non-targeting (control) Accell siRNA or *Serpinh1*-targeting Accell siRNA (Dharmacon, Lafayette, USA). To determine whether pharmacological inhibition affected collagen biosynthesis, we treated slices with 2-hydroxy-3-nitro-5-(phenylmethyl)benzaldehyde (COL003), which is a selective inhibitor of HSP47 (9). Samples were obtained from three independent experiments (biological replicates), which were each carried out in triplicate (technical replicates).

### ATP/Protein

Adenosine triphosphate (ATP) and protein content in slices were determined with an ATP Bioluminescence Kit (Roche Diagnostics, Mannheim, Germany) and RC DC Protein Assay (Bio-Rad, Munich, Germany), respectively (20). Briefly, slices (1/tube) were homogenized in 1 mL of ice-cold sonication solution (70% ethanol and 2 mM EDTA) using a Minibead-beater (2 cycles of 45 s). After centrifugation (16,000 x *g* at 4°C for 5 min), ATP levels in the supernatant were measured. Sample supernatants were subsequently incubated overnight at 37 °C in opened tubes to remove sonication solution through evaporation. Afterwards, upon reconstitution of the pellet, protein levels were determined. ATP values (pmol) were then normalized to the total amount of protein (μg).

### mRNA Expression

Total RNA was isolated from slices using a Maxwell 16 LEV SimplyRNA Tissue Kit (Promega, Leiden, The Netherlands). After confirming the yield and purity with a ND-100 spectrophotometer (NanoDrop Technologies, Wilmington, USA), isolated RNA was reverse transcribed using a Reverse Transcription System Kit (Promega) and thermal cycler (22°C for 10 min, 42°C for 15 min, and 95°C for 5 min). Real-time quantitative polymerase chain reaction (qPCR) was performed using specific primers (Table 1), FastStart Universal SYBR Green Master Mix (Roche, Almere, The Netherlands), and a ViiA7 qPCR machine (Applied Biosystems, Bleiswijk, The Netherlands), which was configured with 1 cycle of 10 min at 95°C and 40 consecutive cycles of 15 s at 95°C, 30 s at 60°C, and 30 s at 72°C. mRNA expression was calculated as fold induction, using *Ywhaz* as a reference gene.

**Table 1:** Primers.

**Gene**	**Protein**	**Forward sequence (5^**′**^ → 3^**′**^)**	**Reverse sequence (5^**′**^ → 3^**′**^)**
*Atf4*	ATF4	AAGGAGGAAGACACTCCCTCT	GTCCATGGGAAGATGTTCTGG
*Acta2*	α-SMA	ACTACTGCCGAGCGTGAGAT	CCAATGAAAGATGGCTGGAA
*Col1a1*	COL1A1	TGACTGGAAGAGCGGAGAGT	ATCCATCGGTCATGCTCTCT
*Edem1*	EDEM1	GGGGCATGTTCGTCTTCGG	CGGCAGTAGATGGGGTTGAG
*Fn*	FN	CGGAGAGAGTGCCCCTACTA	CGATATTGGTGAATCGCAGA
*Hsp90b1*	GRP94	TCGTCAGAGCTGATGATGAAGT	GCGTTTAACCCATCCAACTGAAT
*Hspa5*	BIP	GACTGCTGAGGCGTATTTGG	AGCATCTTTGGTTGCTTGTCG
*Serpine1*	PAI-1	GCCAGATTTATCATCAATGACTGGG	GGAGAGGTGCACATCTTTCTCAAAG
*Serpinh1*	HSP47	AGGTCACCAAGGATGTGGA	CAGCTTCTCCTTCTCGTCGT
*Syvn1*	HRD1	CGTGTGGACTTTATGGAACGC	CGGGTCAGGATGCTGTGATAAG
*Tnfrsf11b*	OPG	ACAGTTTGCCTGGGACCAAA	CTGTGGTGAGGTTCGAGTGG
*Xbp1* (spliced)	XBP1	CTGAGTCCGAATCAGGTGCAG	GTCCATGGGAAGATGTTCTGG
*Ywhaz*	14-3-3ζ	TTACTTGGCCGAGGTTGCT	TGCTGTGACTGGTCCACAAT

### Protein Secretion

Pooled culture medium samples were analyzed using a Mouse Procollagen Type 1 *N*-terminal Propeptide (P1NP) ELISA Kit (Abcam, Cambridge, USA) and Mouse Fibronectin ELISA Kit (Abcam), according to the manufacturer's instructions. Briefly, samples and standards (50 μL/well) as well as antibody cocktail (50 μL/well) were pipetted into a pre-coated 96-well plate, which was subsequently incubated for 60 min at room temperature on a plate shaker set to 500 rpm. After washing the plate 3 times, 3,3′,5,5′-tetramethylbenzidine (TMB) substrate (100 μL/well) was pipetted in each well, and the plate was incubated for 10 min at room temperature on a plate shaker set to 500 rpm. To stop enzymatic conversion of TMB, stop solution (100 μL/well) was added. Optical densities were subsequently measured using a BioTek Synergy HT (BioTek Instruments, Vermont, USA). To account for optical imperfections in the plate, wavelength correction was applied by subtracting readings at 550 nm from readings at 450 nm. P1NP and fibronectin concentrations were interpolated from their respective standard curves. Furthermore, degradation of COL3 by matrix metalloproteinase 9 was analyzed by measuring the concentration of respective degradation fragments (C3M), using a manual competitive ELISA developed by Nordic Bioscience (Herlev, Denmark) (22).

### Protein Expression

Western blotting was used to analyze α-SMA, FKBP65, LC3B, and HSP47 expression in lysate, whereas dot blotting was used to evaluate COL1 trimer content in lysate and culture medium. Lysate was prepared by isolating protein from slices with ice-cold RIPA lysis buffer (Fisher Scientific, Landsmeer, The Netherlands) and a Minibead-beater for homogenization. After centrifuging the lysate (16,000 x *g* at 4°C for 30 min), the supernatant was collected and analyzed to determine the protein concentration. To investigate protein expression through western blotting, protein (10 μg) was heated (75°C for 15 min) and then separated through sodium dodecyl sulfate-polyacrylamide gel electrophoresis (SDS-PAGE), using 10% gels, and blotted onto polyvinylidene fluoride (PVDF) membranes using a Trans-Blot Turbo Transfer System (Bio-Rad). To examine COL1 trimer content by dot blotting, undiluted culture medium samples (2 μL/dot) and diluted protein lysates (2 μg/2μL/dot) were aspirated onto nitrocellulose blotting membranes (Bio-Rad), which were air-dried for 10 min. Regardless of the blotting technique, subsequent membrane processing steps were similar. After blocking in 5% non-fat milk/TBST (Bio-Rad) for 1 h, PVDF and nitrocellulose membranes were incubated overnight (at 4°C) with primary antibodies (Table 2), followed by an incubation with appropriate secondary antibodies for 1 h. Clarity Western ECL blotting substrate (Bio-Rad) and a ChemiDoc Touch Imaging System (Bio-Rad) were used to visualize protein bands/dots. Vinculin (VCL) was used as a loading control for western blotting.

**Table 2:** Antibodies.

**Protein**	**Primary antibody**	**Secondary antibody**
α-SMA	Mouse anti-α-SMA (A2547, 1:5000, Sigma-Aldrich)	Rabbit anti-mouse HRP (P0260, 1:5000, Dako, Santa Clara, USA)
COL1	Rabbit anti-COL1 (ab34710, 1:2000, Abcam)	Goat anti-rabbit HRP (P0448, 1:2000, Dako)
FKBP65	Rabbit anti-FKBP65 (12172-1-AP, 1:500, Proteintech, Manchester, UK)	Goat anti-rabbit HRP (P0448, 1:2000, Dako)
HSP47	Rabbit anti-HSP47 (ab109117, 1:2000, Abcam)	Goat anti-rabbit HRP (P0448, 1:2000, Dako)
LC3B	Rabbit anti-LC3B (ab51520, 1:3000, Abcam)	Goat anti-rabbit HRP (P0448, 1:2000, Dako)
VCL	Mouse anti-VCL (sc-73614, 1:500, Santa Cruz, California, USA)	Rabbit anti-mouse HRP (P0260, 1:5000, Dako)

### Tissue Stainings

Slices were fixed in formalin (4%) at 4°C for 24 h, after which they were dehydrated in graded ethanol baths, cleared in xylene, and embedded horizontally in paraffin. Before staining, sections (4 μm) were deparaffinized in xylene and rehydrated in graded ethanol baths. Tissue morphology was investigated with a routine hematoxylin and eosin (H&E) staining and fibrillar COL1 and COL3 networks were visualized using a Picro Sirius Red Stain Kit (Abcam). High-resolution digital data was then obtained by scanning stained sections with a C9600 NanoZoomer (Hamamatsu Photonics, Hamamatsu, Japan). Semi-quantitative tissue damage scores were assigned to H&E stained sections using our previously published scoring system (23). The extent of collagen deposition was estimated by visual inspection of picrosirius red-stained sections using unpolarized light.

### Statistics

GraphPad Prism (version 8.0) was used to analyze data with a two-way analysis of variance (ANOVA) followed by Bonferroni's multiple comparisons test. Differences were considered to be statistically significant when *p* < 0.05.

## Results

### TGFβ1 Did Not Affect the Viability and Morphology of Slices

**Figure 1:**
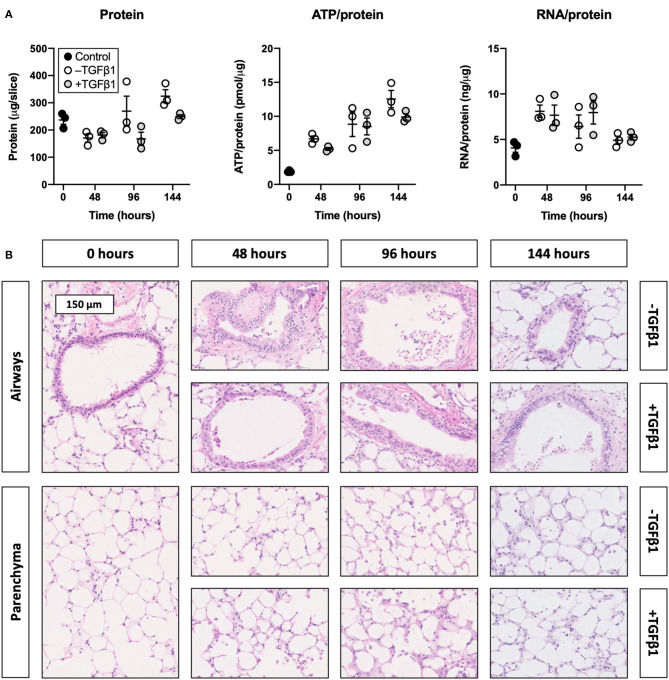
Effect of TGFβ1 on the viability and morphology of slices. Slices were sampled after slicing (0 h) and after 48, 96, or 144 h of incubation without or with 5 ng/mL TGFβ1 (*n* = 3). Protein, ATP/protein, and RNA/protein content **(A)** were analyzed to assess the viability, and HE stainings were performed to investigate the morphology **(B)**. Values represent individual experiments performed in triplicate and are accompanied with the arithmetic mean (horizontal line) ± standard error of the mean (error bars).

To determine whether slices remained viable upon exposure to TGFβ1, we analyzed the protein, ATP/protein, and RNA/protein content as well as the morphology (Figure 1). As shown, TGFβ1 did not significantly change the protein, ATP/protein, and RNA/protein content. Regardless of whether TGFβ1 was added to culture medium, the protein content remained fairly stable over time, whereas the ATP/protein content gradually increased. The RNA/protein content, however, was marked by an initial increase after 48 h of incubation, after which it gradually decreased to the same levels observed at 0 h. Furthermore, TGFβ1 did not affect the morphology of slices (tissue damage scores are shown in Supplementary Figure 1), and the overall morphology remained sufficiently preserved for up to 144 h because only moderate tissue damage was observed.

### TGFβ1 Augmented the Development of Fibrogenesis in Slices

**Figure 2:**
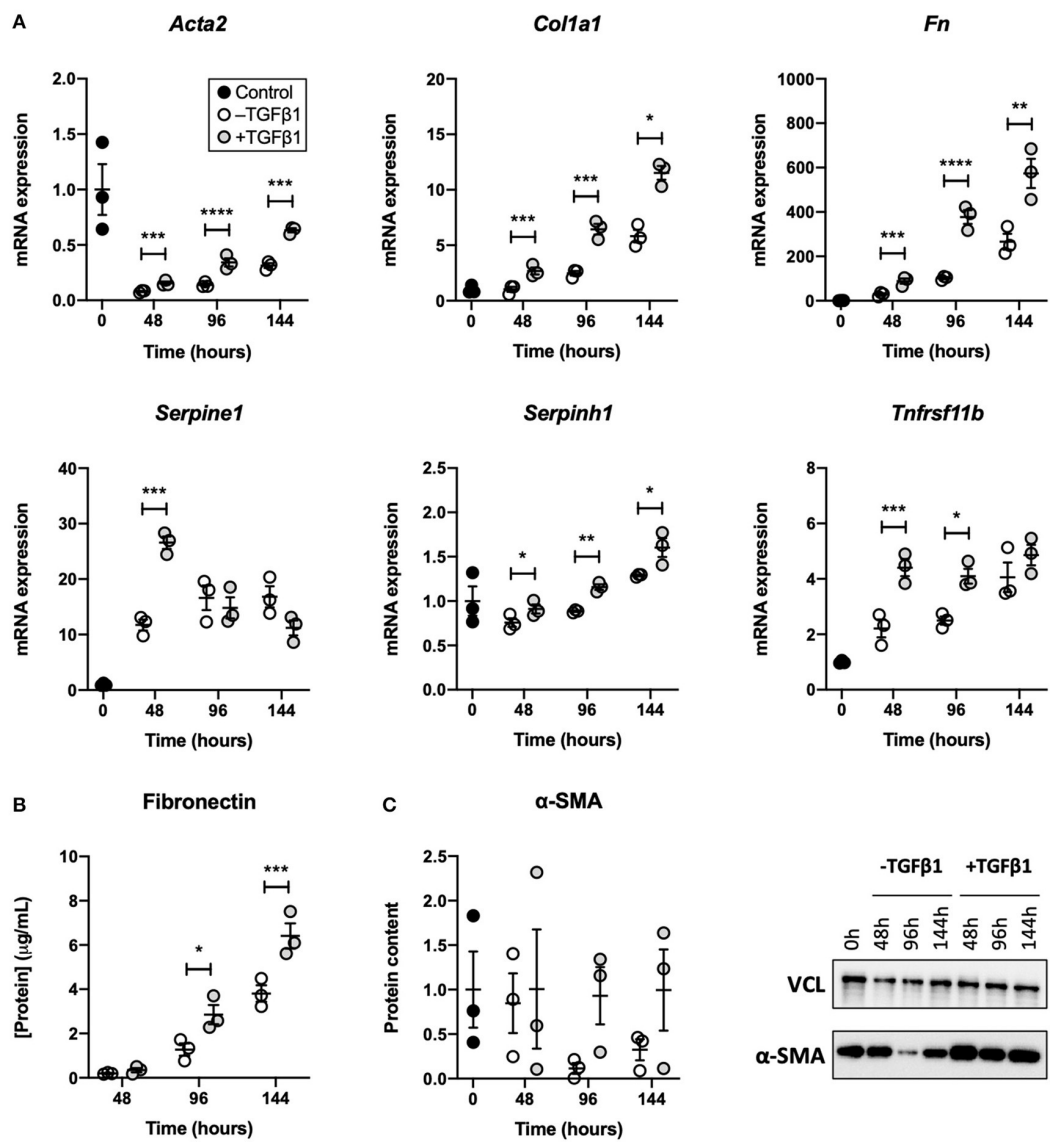
Effect of TGFβ1 on the development of fibrogenesis in slices. Slices were collected after slicing (0 h) and after 48, 96, or 144 h of incubation without or with 5 ng/mL TGFβ1 (*n* = 3). Expression of fibrogenesis-related genes **(A)**, secretion of fibronectin into culture medium **(B)**, and expression of α-SMA **(C)** were analyzed to examine the development of fibrogenesis. Values depict individual experiments performed in triplicate and are accompanied with the arithmetic mean (horizontal line) ± standard error of the mean (error bars). (*p **<** 0.05, **p **<** 0.01, ***p **<** 0.001, and ****p **<** 0.0001).

To establish whether TGFβ1 augmented fibrogenesis in slices, we measured the expression of fibrogenesis-related genes, secretion of fibronectin into culture medium, and expression of α-SMA (Figure 2). As demonstrated, TGFβ1 significantly increased mRNA expression of all analyzed fibrogenesis-related genes. Over time, mRNA expression of *Col1a1, Fn, Serpine1, Serpinh1*, and *Tnfrsf11b* steadily increased, whereas mRNA expression of *Acta2* initially decreased but later increased. Treating slices with TGFβ1 also enhanced the secretion of fibronectin into culture medium. Additionally, in slices cultured without TGFβ1, α-SMA expression declined, albeit not significantly. Culturing slices with TGFβ1, however, resulted in stable α-SMA expression over time.

### TGFβ1 Promoted Collagen Secretion and Deposition in Slices

**Figure 3:**
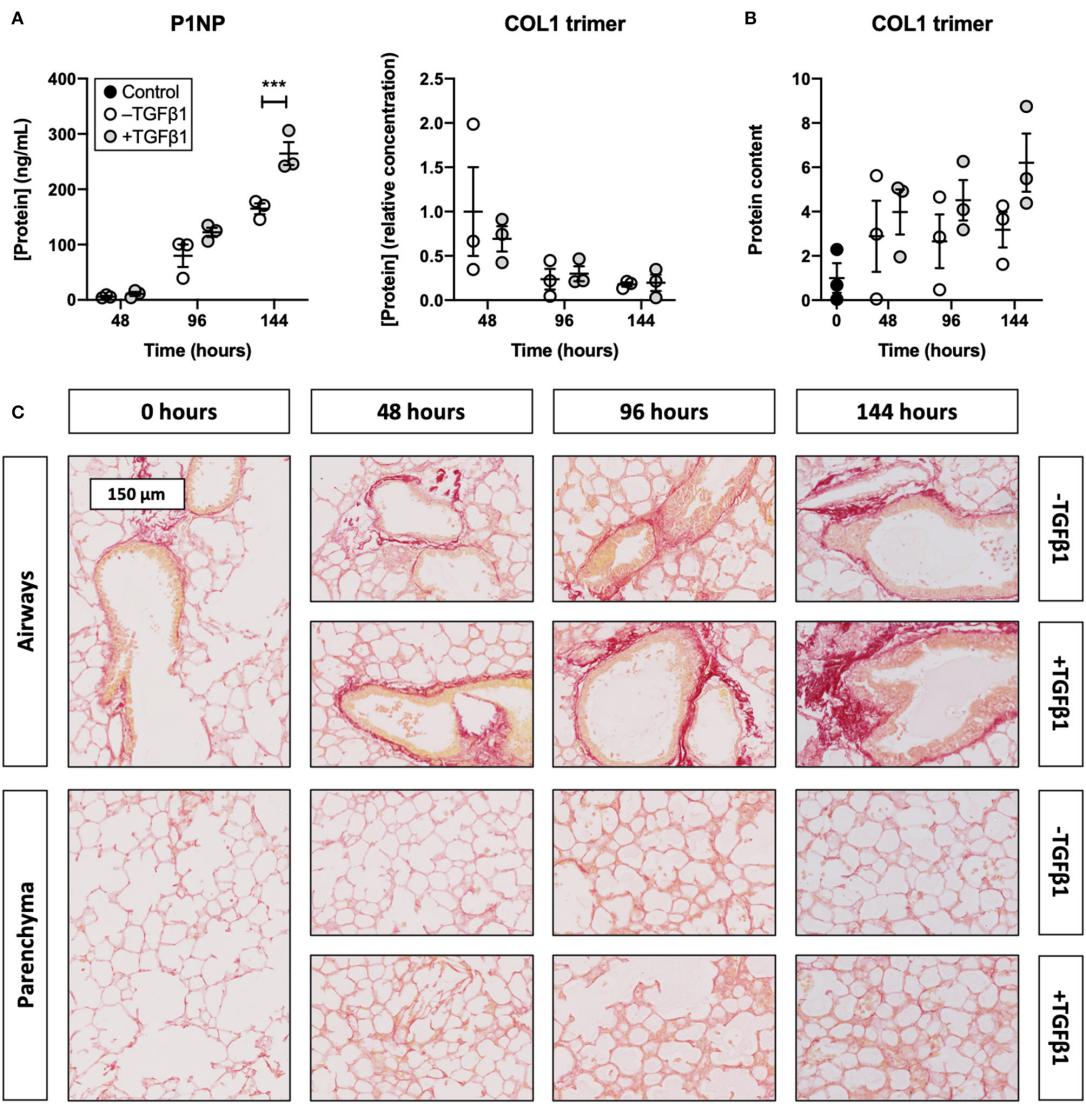
Effect of TGFβ1 on collagen secretion and deposition in slices. Slices were sampled after slicing (0 h) and after 48, 96, or 144 h of incubation without or with 5 ng/mL TGFβ1 (*n* = 3). The concentration of P1NP and trimeric COL1 in culture medium **(A)** was measured to study collagen secretion, and COL1 trimer content in lysate **(B)** was investigated to track its incorporation into the ECM. Networks of COL1 and COL3 were visualized **(C)** with picrosirius red stainings. Values depict individual experiments performed in triplicate and are accompanied with the arithmetic mean (horizontal line) ± standard error of the mean (error bars). (***p **<** 0.001).

To identify whether TGFβ1 enhanced collagen biosynthesis in slices, we measured the concentration of P1NP and trimeric COL1 in culture medium as well as the incorporation of COL1 trimers into the ECM (Figure 3). As illustrated, the P1NP concentration gradually increased, and treatment with TGFβ1 further boosted this increase after 144 h of incubation. In contrast, the concentration of COL1 trimers in culture medium steadily declined and was not affected by TGFβ1. COL1 trimer content in lysate, however, increased over time and seemed to further increase, albeit not significantly, after exposing slices to TGFβ1. Similar trends were observed in sections stained with picrosirius red as networks of fibrillar COL1 and COL3 became more prominent.

### Accell siRNA Did Not Affect the Viability and Morphology of Slices

**Figure 4:**
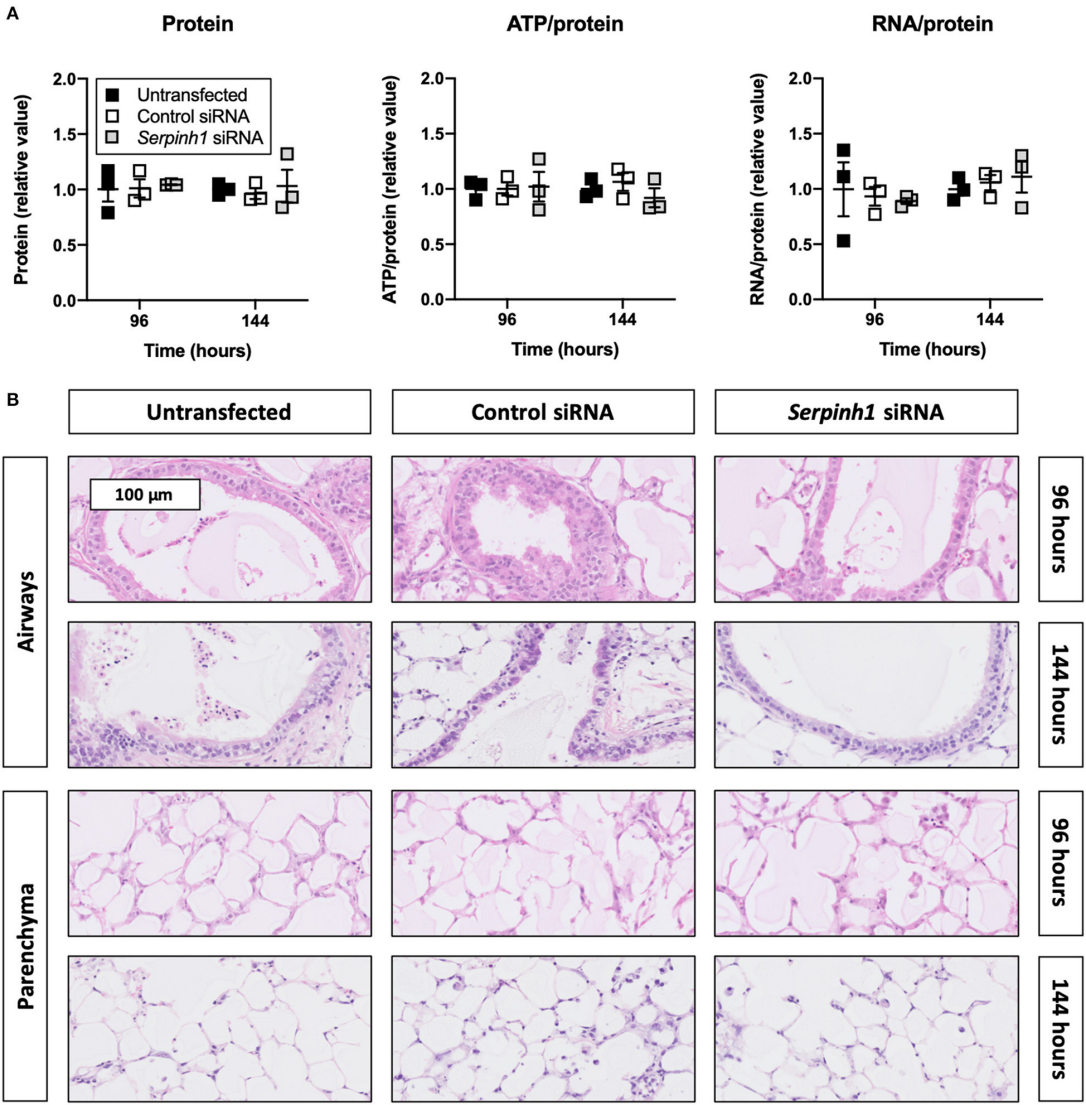
Effect of Accell siRNA on the viability and morphology of slices. Untransfected and transfected slices were collected after 96 or 144 h of incubation with 5 ng/mL TGFβ1 (*n* = 3). Protein, ATP/protein, and RNA/protein content **(A)** were measured to study the viability, and HE stainings were conducted to assess the morphology **(B)**. Values represent individual experiments performed in triplicate and are accompanied with the arithmetic mean (horizontal line) ± standard error of the mean (error bars).

To check whether Accell siRNA induced toxic effects in TGFβ1-treated slices, we assessed protein, ATP/protein, and RNA/protein content as well as the morphology (Figure 4). As displayed, Accell siRNA did not significantly alter protein, ATP/protein, and RNA/protein content in slices. Similarly, no noticeable differences were observed in the morphology of untransfected and transfected slices (tissue damage scores are shown in Supplementary Figure 2).

### Accell siRNA Induced Knockdown of mRNA and Protein in Slices

**Figure 5:**
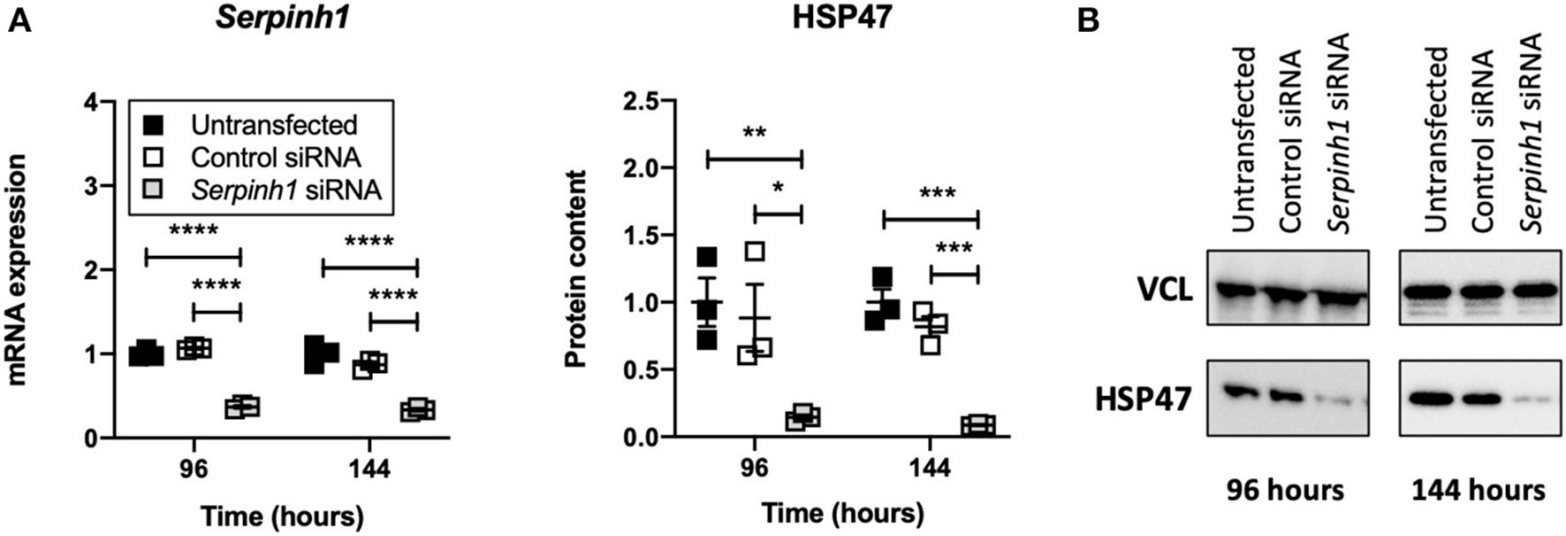
Effect of Accell siRNA on mRNA and protein knockdown in slices. Untransfected and transfected slices were sampled after 96 or 144 h of incubation with 5 ng/mL TGFβ1 (*n* = 3). Expression of *Serpinh1* mRNA **(A)** and its respective protein HSP47 **(B)** were analyzed to verify whether Accell siRNA induced RNA interference. Values represent individual experiments performed in triplicate and are accompanied with the arithmetic mean (horizontal line) ± standard error of the mean (error bars). (*p **<** 0.05, **p **<** 0.01, ***p **<** 0.001, and ****p **<** 0.0001).

To study whether Accell siRNA induced RNA interference in slices treated with TGFβ1, we examined expression of *Serpinh1* mRNA and its respective protein HSP47 (Figure 5). As depicted, significant mRNA (~65%) and protein (~90%) knockdown was observed in slices that were treated with *Serpinh1*-targeting siRNA for 96 and 144 h. Non-targeting (control) siRNA did not cause non-specific knockdown of mRNA and protein levels.

### HSP47 Knockdown Altered the Secretion of Fibronectin by Slices

**Figure 6:**
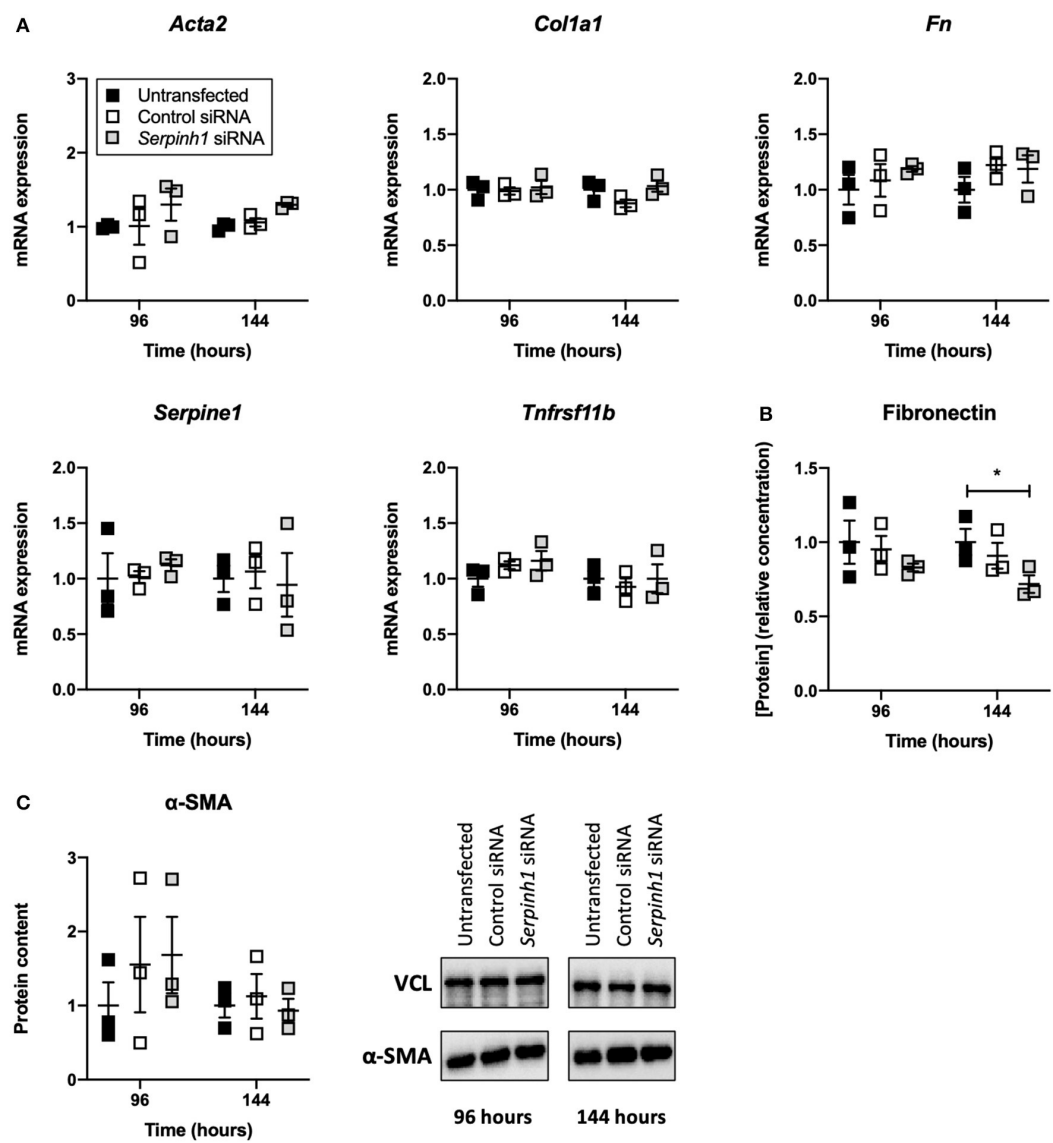
Effect of HSP47 knockdown on the development of fibrogenesis in slices. Untransfected and transfected slices were collected after 96 or 144 h of incubation with 5 ng/mL TGFβ1 (*n* = 3). Expression of fibrogenesis-related genes **(A)**, secretion of fibronectin into culture medium **(B)**, and expression of α-SMA **(C)** were measured to identify potential downstream effects on fibrogenesis after knockdown of HSP47. Values represent individual experiments performed in triplicate and are accompanied with the arithmetic mean (horizontal line) ± standard error of the mean (error bars). (*p **<** 0.05).

To study whether knockdown of HSP47 affected fibrogenesis in TGFβ1-treated slices, we measured the expression of fibrogenesis-related genes, secretion of fibronectin into culture medium, and expression of α-SMA (Figure 6). As shown, no significant differences were observed between untransfected and transfected slices with respect to the expression of fibrogenesis-related genes and α-SMA. However, in comparison to untransfected slices, slices treated with *Serpinh1*-targeting siRNA displayed a significantly reduced (~30%) secretion of fibronectin into culture medium after 144 h of incubation.

### HSP47 Knockdown Did Not Diminish Collagen Secretion and Deposition in Slices

**Figure 7:**
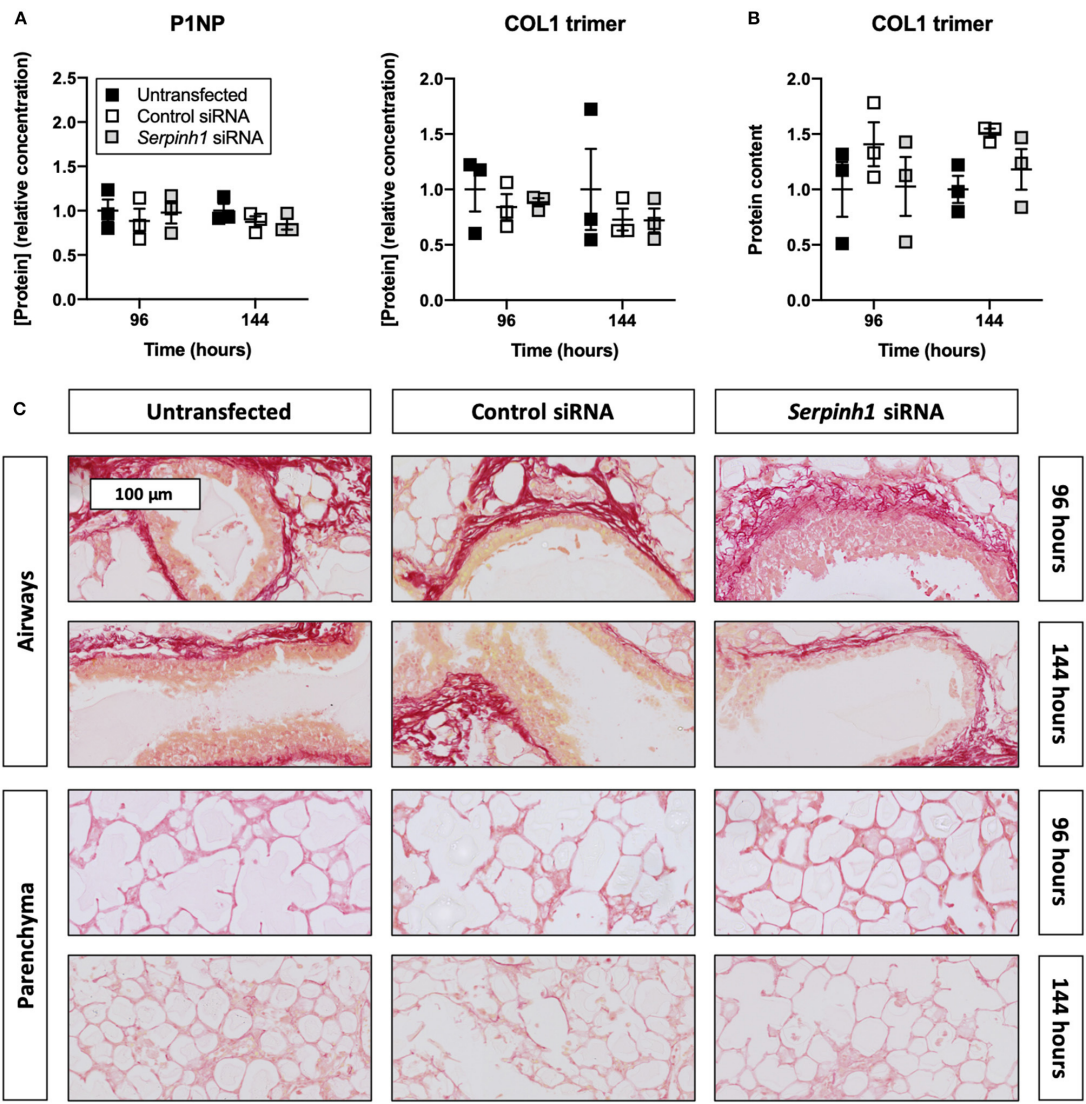
Effect of HSP47 knockdown on collagen secretion and deposition in slices. Untransfected and transfected slices were sampled after 96 or 144 h of incubation with 5 ng/mL TGFβ1 (*n* = 3). The concentration of P1NP and trimeric COL1 in culture medium **(A)** was measured to identify downstream effects on collagen secretion, and COL1 trimer content in lysate **(B)** was assessed to monitor its incorporation into the ECM. Networks of COL1 and COL3 were visualized **(C)** using picrosirius red stainings. Values depict individual experiments performed in triplicate and are accompanied with the arithmetic mean (horizontal line) ± standard error of the mean (error bars).

To determine whether knockdown of HSP47 affected the biosynthesis of collagen in slices, we measured the concentration of P1NP and trimeric COL1 in culture medium as well as the incorporation of COL1 trimers into the ECM (Figure 7). As demonstrated, no significant differences were observed between untransfected and transfected slices regarding the concentration of P1NP and trimeric COL1 in culture medium; nor was the incorporation of COL1 trimers into the ECM affected. Likewise, the formation of fibrillar COL1 and COL3 networks remained unaffected upon knockdown of HSP47.

## Discussion

**Figure 8:**
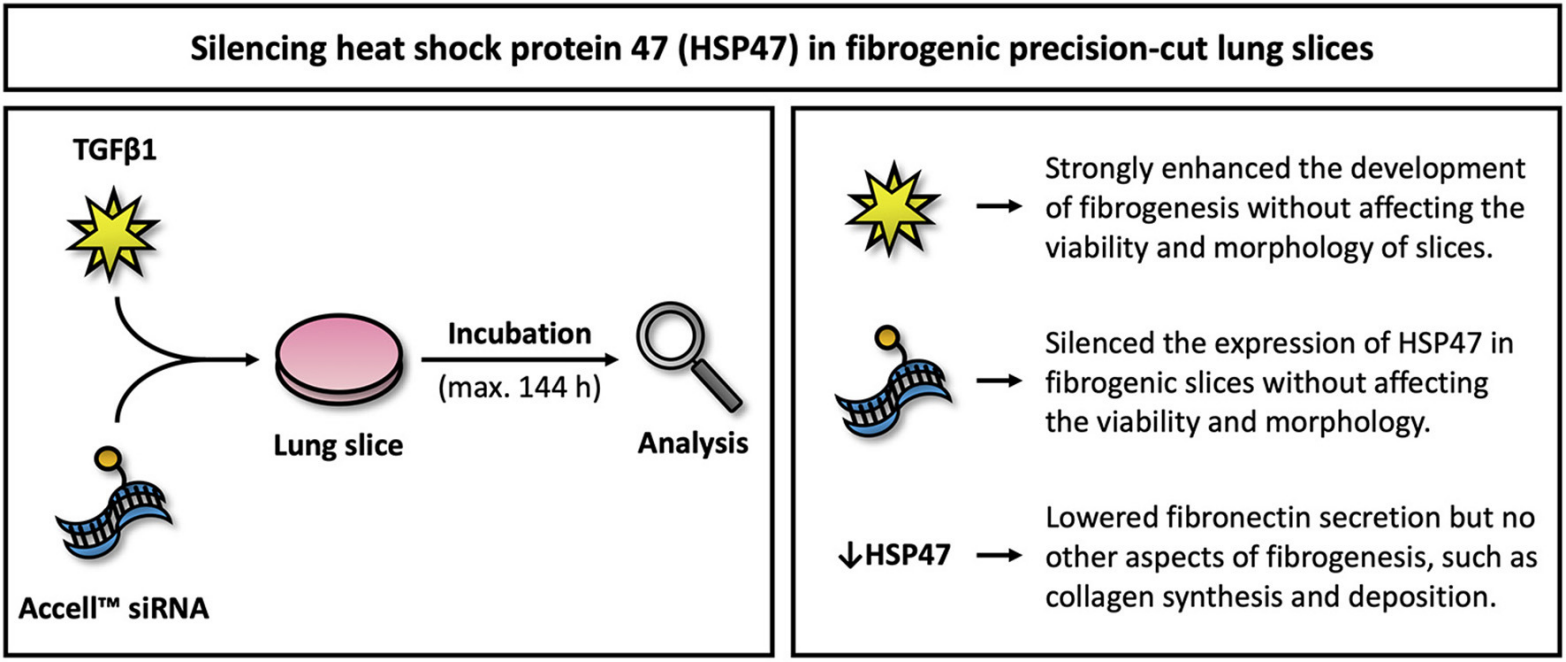
Overview of key findings. This figure illustrates key findings of this study. Further studies are warranted to elucidate downstream effects on fibrosis upon silencing HSP47.

The main objective of this study was to evaluate the therapeutic potential of *Serpinh1*-targeting siRNA in fibrogenic lung slices (Figure 8). Our study demonstrated that slices remained viable for up to 144 h of incubation, even when treated with TGFβ1 and Accell siRNA. In addition, TGFβ1 was shown to augment fibrogenesis as well as collagen secretion and deposition. We also observed specific and significant knockdown of HSP47 (~90%) after culturing slices with *Serpinh1*-targeting siRNA for 96 or 144 h. Knockdown of HSP47, however, only affected the secretion of fibronectin into culture medium but no other aspects of fibrogenesis, such as mRNA expression of fibrogenesis-related genes, expression of α-SMA, and secretion of collagen as well as its incorporation into the ECM.

First of all, we analyzed protein, ATP/protein, and RNA/protein content as well as the morphology to unravel whether slices remained viable upon exposure to TGFβ1. In general, slices were viable and their morphology remained sufficiently preserved for up to 144 h in the presence of TGFβ1. Similar observations have been made for rat lung slices when they were cultured for 72 h with 10 ng/mL TGFβ1 as no differences in ATP/protein content were detected (24). Another study demonstrated human lung slices also maintained their mitochondrial activity and morphology for up to 120 h when treated with a “fibrosis cocktail,” which was composed of 5 ng/mL TGFβ1, 5 μM platelet-derived growth factor AB, 10 ng/mL tumor necrosis factor alpha, and 5 μM lysophosphatidic acid (25). Though not strictly classifiable as precision-cut lung slices, cubic human lung explants (2 mm^3^) were also recently shown to remain viable for up to 7 days of incubation when cultured in medium containing 10 ng/mL TGFβ1 (26). Nevertheless, due to the pleiotropic nature of TGFβ1, it cannot be ruled out that other aspects of viability were affected (e.g., cell death and/or proliferation). These findings should therefore be interpreted carefully.

After confirming that slices remained viable, we studied whether TGFβ1 augmented fibrogenesis. As demonstrated, mRNA expression of *Acta2, Col1a1, Fn, Serpine1, Serpinh1*, and *Tnfrsf11b* was clearly increased by TGFβ1, which controls a wide range of processes related to fibrosis (27). The initial decline of *Acta2* mRNA expression in slices that were not treated with TGFβ1 cannot be readily explained but we speculate it was caused by a loss of vascular smooth muscle cells because they also express this gene (28). Remarkably, in our current study, we observed a much greater induction of fibrogenesis-related genes than in our previous study (20). This can be explained by differences in incubator oxygen concentration; slices cultured at 20% O_2_ (current study) are considerably more viable than slices incubated at 80% O_2_ (previous study) (23). Secretion of fibronectin, which is a glycoprotein that connects ECM proteins to cells via integrins, was also increased upon exposure to TGFβ1 (29). Though we did not investigate the localization of fibronectin deposition, others have shown its deposition is generally more abundant in the outermost region of lung slices (25). Lastly, TGFβ1 appeared to only affect α-SMA expression after 96 h of incubation, albeit not significantly—perhaps, our slice incubation time was too short to reveal significant differences.

We then assessed whether TGFβ1 affected the concentration of P1NP and COL1 trimers in culture medium, the incorporation of COL1 trimers into the ECM, and the formation of fibrillar COL1 and COL3 networks. In slices, the overall production of COL1 clearly increased over time and was further enhanced by TGFβ1, as demonstrated by P1NP ELISA. At first glance, this finding seems to contradict the steadily declining COL1 trimer concentration in the medium. However, this discrepancy can be explained by differences between the immunogens that were used for raising respective antibodies. The P1NP ELISA made use of antibodies raised against the *N*-terminal propeptide chains of COL1, whereas the COL1 trimer antibody was raised against trimeric COL1 with a triple-helix structure. Because the P1NP ELISA cannot differentiate between cleaved or uncleaved procollagens, these findings suggest COL1 trimers were misfolded (rendering epitopes unrecognizable) and/or incorporated into the ECM. The latter most likely occurred as COL1 trimer content in lysate was increased and fibrillar COL1 and COL3 networks became more prominent, as revealed by picrosirius red stainings.

Next, we validated whether our previously published transfection method could still be used to achieve mRNA and protein knockdown without affecting the viability and morphology of slices (19, 20). This was necessary because different incubation conditions were used in our current study (i.e., incubator oxygen concentration, culture medium composition, and presence of TGFβ1). As hypothesized, non-targeting and *Serpinh1*-targeting siRNA did not affect the viability or morphology of slices. Furthermore, *Serpinh1*-targeting siRNA induced significant mRNA and protein knockdown. These findings correlate well with our previous study and illustrate the usefulness of Accell siRNA to trigger RNA interference in slices (20). Given that slices used in this study were more viable than those used in our previous study, we should consider our current findings as more relevant (i.e., slices represented the cellular microenvironment more accurately). Although other aspects of viability were not evaluated, sufficient evidence was collected to confirm Accell siRNA can be used to study the effects of *Serpinh1*-targeting siRNA in fibrogenic slices.

After confirming successful knockdown of HSP47, we found that neither the expression of fibrogenesis-related genes nor expression of α-SMA was affected. Aside from contradicting our previously published results (i.e., HSP47 knockdown lowered *Serpine1* and *Tnfrsf11b* mRNA expression in slices), our current findings also contradict other published studies that showed knockdown of HSP47 rapidly lowered mRNA expression of *Acta2* and *Col1a1* in mouse dermal fibroblasts, hepatic stellate cells, and embryo fibroblasts (17, 20, 30). In those studies, the quick onset of effects *in vitro* (<24 h) was achieved by using alternative transfection techniques. Cationic lipid-based transfection reagents, for instance, are typically taken up by cells very quickly (within a few hours), whereas uptake of Accell siRNA requires more time (20). Furthermore, as matrix stiffness regulates cell behavior, it cannot be ruled out that myofibroblasts cultured on plastic were phenotypically different from those in slices (31). Surprisingly, the secretion of fibronectin was lowered after knockdown of HSP47. This effect is not fully understood because, in mouse embryo fibroblasts, HSP47 has been shown not to interact directly with fibronectin or to affect its secretion (32).

Lastly, we studied whether collagen secretion and deposition were diminished in slices upon treatment with *Serpinh1*-targeting siRNA. Surprisingly, we did not identify any effects on collagen biosynthesis. Because HSP47 has been previously demonstrated to be essential for the formation of special secretory vesicles, it might have been the case that residual HSP47 (10%) was sufficient to enable the secretion of procollagens (32). This hypothesis could be tested using slices prepared from tissue of conditional HSP47-knockout mice. Furthermore, these findings contrast not only published *in vitro* studies but also *in vivo* studies; antifibrotic effects of *Serpinh1*-targeting siRNA have been previously observed in various fibrosis models, such as pulmonary, hepatic, renal, peritoneal, and dermal fibrosis (12–17, 32, 33). These studies, however, did not provide mechanistic insights into the effects over time. Effects on collagen biosynthesis were only determined after 3 or 4 weeks of (frequent) siRNA administration. The source of therapeutic effects in animals is therefore not entirely clear. Knockdown of HSP47 may have affected fibrosis through several, non-mutually exclusive mechanisms. Identifying these mechanisms will help us to better understand the therapeutic potential of *Serpinh1*-targeting siRNA. The use of advanced analytical techniques, such as single-cell sequencing, could help us to detect and characterize affected molecular mechanisms.

To gain more insight into these mechanisms, we explored whether HSP47 knockdown affected ER stress, autophagy, collagen processing, and ECM degradation (Supplementary Figure 3). As ER stress may have developed due to the accumulation of misfolded collagens, we measured mRNA expression of genes related to the unfolded protein response (UPR)—an evolutionary conserved stress response that becomes activated when unfolded and misfolded proteins accumulate in the ER (34). Knockdown of HSP47, however, did not significantly change mRNA expression of UPR-related genes in slices. We also studied whether autophagy was induced, but detected no significant increase in LC3B after silencing HSP47. Similarly, knockdown of HSP47 did not affect the expression of FKBP65, which interacts with HSP47 during the biosynthesis of collagens to facilitate their stabilization. Instead of solely affecting collagen biosynthesis, knockdown of HSP47 may have also caused therapeutic effects in animals by favoring the incorporation of misfolded collagens, making the ECM more vulnerable to degradation by matrix metalloproteinases. Nevertheless, we did not observe an increased degradation of COL3 in slices.

In addition to using siRNA, we also investigated whether COL003, which is a selective pharmacological inhibitor of HSP47, affected collagen biosynthesis in slices (Supplementary Figure 4). This compound has been previously shown to competitively inhibit interactions between HSP47 and procollagens in mouse embryo fibroblasts, thereby reducing collagen secretion (9, 35). In slices, however, the secretion of collagen was not affected by COL003 at its maximum tolerable concentration (5.0 μM); hence we did not conduct follow-up analyses regarding potential therapeutic effects or mechanisms. COL003 was also considerably more toxic in slices than in mouse embryo fibroblasts, which remained viable when cultured with 100 μM of COL003 (9). This discrepancy is probably caused by the fact that lung slices express biotransformation enzymes, which may have generated toxic metabolites (18). Safer and more effective analogs are therefore greatly desired to determine whether HSP47 is a suitable target for treating fibrosis.

## Conclusion

The aim of this study was to evaluate the therapeutic potential of *Serpinh1*-targeting siRNA in fibrogenic lung slices. First, we demonstrated that fibrogenesis in slices could be augmented by TGFβ1 without affecting their viability and morphology. We subsequently showed that slices with a fibrogenic phenotype were successfully transfected with Accell siRNA, resulting in specific mRNA and protein knockdown. Surprisingly, upon knockdown of HSP47, the secretion of fibronectin was reduced while other aspects of fibrogenesis remained unaffected (e.g., differentiation of fibroblasts into myofibroblasts as well as collagen secretion and deposition). These observations are surprising as others have demonstrated that *Serpinh1*-targeting siRNA attenuated collagen deposition in animals when treated for 3 to 4 weeks. Although the exact source of therapeutic effects in animals remains unclear, the prospect of having a potential target for treating IPF should serve as a strong incentive for future research on HSP47.

## Data Availability Statement

The raw data supporting the conclusions of this article will be made available by the authors, without undue reservation.

## Ethics Statement

The animal study was reviewed and approved by the Dutch Central Authority for Scientific Procedures on Animals.

## Author Contributions

MR, DL, JS, HF, WH, and PO contributed to the conception and design of this study. MR, KE, and JS carried out experiments and analyses. MR, HF, WH, and PO wrote the first draft of the manuscript. All authors contributed to manuscript revision, read, and approved the submitted version.

## Conflict of Interest

The authors declare that the research was conducted in the absence of any commercial or financial relationships that could be construed as a potential conflict of interest.

## Abbreviations

α-SMA: alpha smooth muscle actin
*Acta2*: alpha smooth muscle actin
ANOVA: analysis of variance
*Atf4*: activating transcription factor 4
ATP: adenosine triphosphate
C3M: degradation fragment of collagen type 3
COL003: 2-hydroxy-3-nitro-5-(phenylmethyl)benzaldehyde
COL1: collagen type 1
*Col1a1*: collagen type 1 alpha 1
COL3: collagen type 3
ECM: extracellular matrix
*Edem1*: ER degradation enhancer mannosidase alpha-like 1
ER: endoplasmic reticulum
*Fn*: fibronectin
FKBP65: FK506 binding protein 10
H&E: hematoxylin and eosin
HSP47: heat shock protein 47
*Hsp90b1*: heat shock protein 90 beta family member 1
*Hspa5*: heat shock protein family A member 5
IPF: idiopathic pulmonary fibrosis
LC3B: microtubule-associated protein 1 light chain 3 beta
mRNA: messenger RNA
P1NP: procollagen type 1 *N*-terminal propeptide
PVDF: polyvinylidene fluoride
qPCR: real-time quantitative polymerase chain reaction
RNAi: RNA interference
SDS-PAGE: sodium dodecyl sulfate-polyacrylamide gel electrophoresis
*Serpine1*: serine (or cysteine) peptidase inhibitor clade E member 1
*Serpinh1*: serine (or cysteine) peptidase inhibitor clade H member 1
siRNA: small interfering RNA
*Syvn1*: synoviolin 1
TGFβ1: transforming growth factor β1
TMB: 3,3′,5,5′-tetramethylbenzidine
*Tnfrsf11b*: tumor necrosis factor receptor superfamily member 11b
UPR: unfolded protein response
UW: university of Wisconsin
VCL: vinculin
*Xbp1*: x-box binding protein 1
*Ywhaz*: tyrosine 3-monooxygenase activation protein, zeta polypeptide.
