# Supplementary material for: Silencing Heat Shock Protein 47 (HSP47) in Fibrogenic Precision-Cut Lung Slices: A Surprising Lack of Effects on Fibrogenesis?
Source: Front Med (Lausanne). 2021 Feb 15;8:607962. doi: 10.3389/fmed.2021.607962 (PMC7917123; doi:10.3389/fmed.2021.607962)
Supplement: Supplementary file 1 [file Data_Sheet_1.pdf]

Supplementary information

Silencing heat shock protein 47 (HSP47) in fibrogenic precision-cut lung slices: a surprising lack of effects on fibrogenesis?

Mitchel J.R. Ruigrok[1], Khaled E.M. El Amasi[1], Diana J. Leeming[2], Jannie M.B. Sand[2], Henderik W. Frijlink[1], Wouter L.J. Hinrichs[1,3], Peter Olinga[1].

[1]University of Groningen; Groningen Research Institute of Pharmacy; Department of Pharmaceutical Technology and Biopharmacy; Antonius Deusinglaan 1; 9713 AV Groningen; The Netherlands.

[2]Nordic Bioscience; Herlev Hovedgade 205-207; 2703 Herlev; Denmark.

[3]Corresponding author (phone number: +31 (0)50 36 32398, e-mail address: w.l.j.hinrichs@rug.nl).

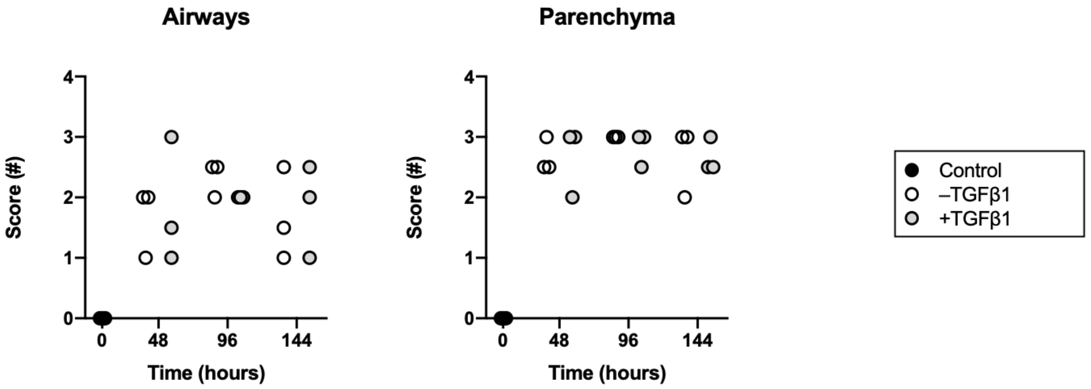

Supplementary figure 1. Effect of TGFβ1 on the morphology of slices. Slices were collected after slicing (0 h) and after 48, 96, or 144 h of incubation without or with 5 ng/mL TGFβ1 (n = 3). To determine whether TGFβ1 enhanced tissue damage, we assigned semi-quantitative scores to H&E stained sections. Values represent individual experiments performed in triplicate.

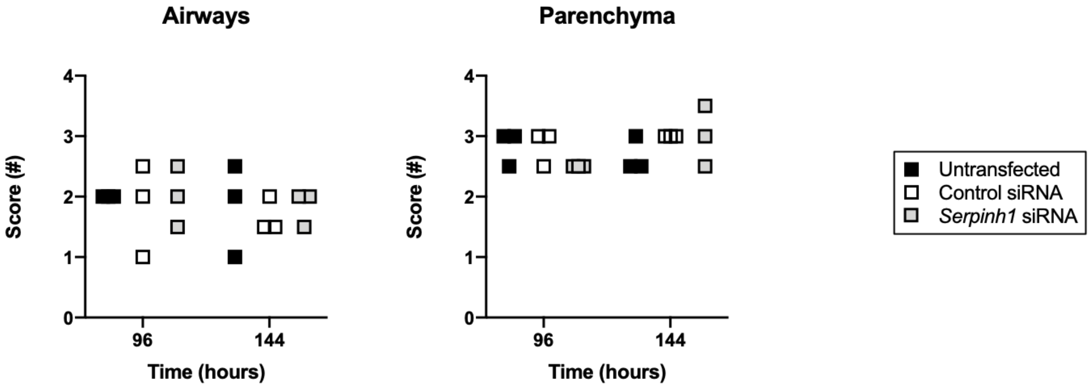

Supplementary figure 2. Effect of Accell siRNA on the morphology of slices. Untransfected and transfected slices were sampled after 96 or 144 h of incubation with 5 ng/mL TGFβ1 (n = 3). To determine whether Accell siRNA enhanced tissue damage, we assigned semi-quantitative scores to H&E stained sections. Values represent individual experiments performed in triplicate.

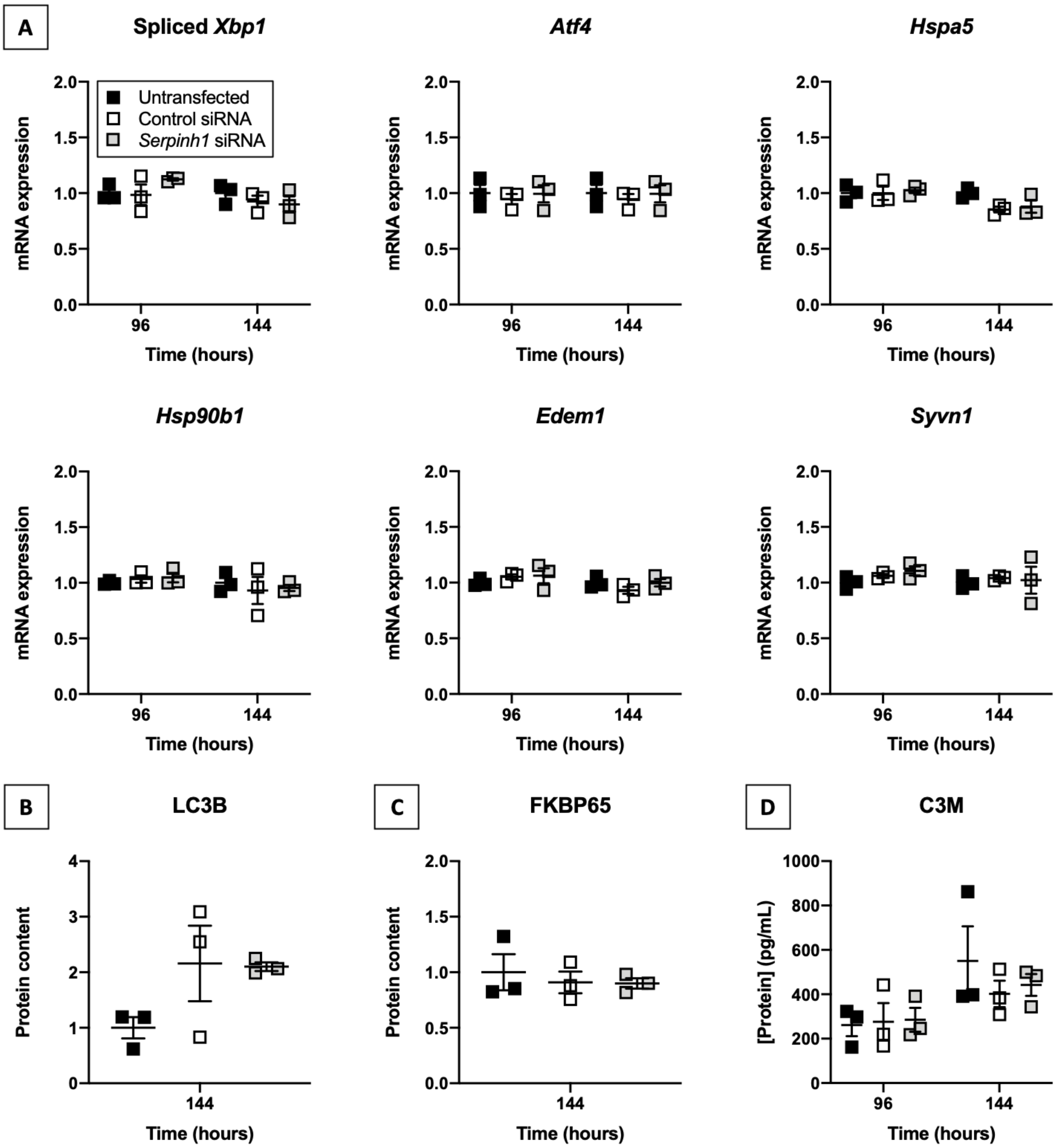

Supplementary figure 3. Effect of HSP47 knockdown on alternative processes. Untransfected and transfected slices were collected after 96 or 144 h of incubation with 5 ng/mL TGFβ1 (n = 3). To identify whether slices displayed ER stress, we measured mRNA expression of UPR-related genes (a). We also analyzed LC3B (b) and FKBP65 (c) expression to investigate whether knockdown of HSP47 affected autophagy or collagen processing, respectively. To examine whether degradation of COL3 was enhanced upon silencing HSP47, we determined C3M levels in culture medium (d). Values depict individual experiments performed in triplicate and are accompanied with the arithmetic mean (horizontal line) ± standard error of the mean (error bars).

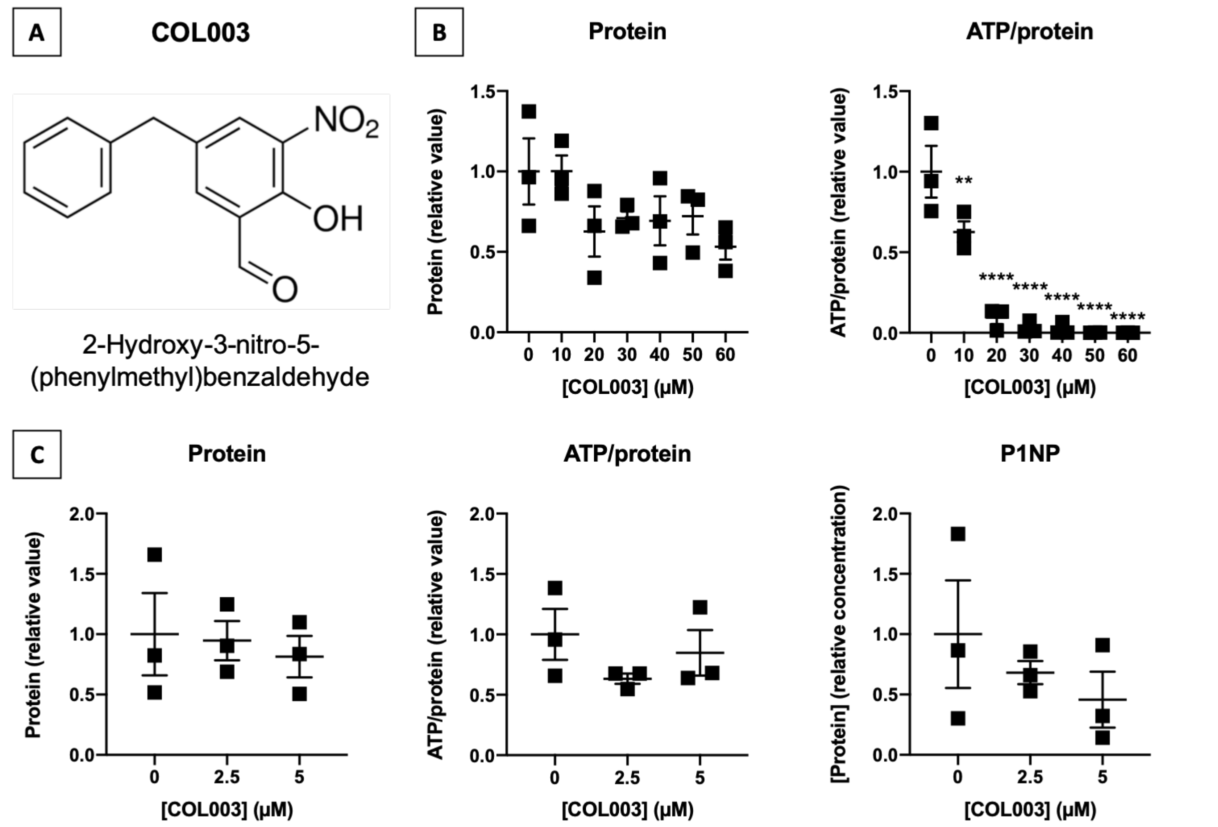

Supplementary figure 4. Effect of COL003 on lung slices. As an alternative to using siRNA, we explored the effects of COL003 (a). We first constructed a dose-response curve (b), based on protein and ATP/protein content, to establish safe concentrations of COL003 in slices that were cultured for 48 hours without TGFβ1 (*n* = 3). Due to COL003 its toxicity, we constructed a second dose-response curve (c) to establish safe concentrations of COL003 in slices that were cultured for 48 hours with 5 ng/mL TGFβ1 (*n* = 3). At its maximum tolerable concentration (5 µM), COL003 did not significantly affect the secretion of P1NP. Values represent individual experiments performed in triplicate and are accompanied with the arithmetic mean (horizontal line) ± standard error of the mean (error bars). (** $p < .01$ and **** $p < .0001$)
